# Dietary Puerarin Supplementation Improves Immune Response and Antioxidant Capacity of Sows

**DOI:** 10.3390/antiox13030290

**Published:** 2024-02-27

**Authors:** Shanchuan Cao, Xinglai Li, Heng Yin, Juan Wang, Jingbo Liu

**Affiliations:** 1 School of Life Science and Engineering, Southwest University of Science and Technology, Mianyang 621010, China; 2 Department of Animal Resource and Science, Dankook University, Cheonan 31116, Republic of Korea

**Keywords:** reproductive performance, colostrum, milk, cytokines, antioxidant parameters, sow, puerarin

## Abstract

Puerarin is an isoflavone extracted from *Pueraria mirifica*, a wildlife leguminous plant. It has been reported to possess antioxidant, anti-inflammatory, and anti-bacterial properties. However, the effects of directly adding puerarin to the diets of sows, in terms of reproductive performance and antioxidant properties, have not been reported. For this study, 240 sows with varying parities were selected and randomly divided into six treatment groups using a two × three experimental design. The six treatment groups consisted of two diets (control and puerarin) and three parities (zero, one, and two parities or more). The puerarin group was supplemented with 1 g/kg of puerarin. The experiment commenced with mating and continued until 21 days post-delivery. The sow reproductive performance was not affected by supplementing their diets with puerarin (*p* > 0.05). Dietary supplementation with puerarin significantly increased the daily body weight (BW) gain of piglets and their mean BW at weaning (*p* < 0.05). Compared with the control group, sows in the puerarin group had significantly higher glutathione peroxidase activity in serum and also significantly increased immunoglobulin A and G levels in serum, colostrum, and milk, but significantly lower malondialdehyde concentration in serum (*p* < 0.05). Thus, puerarin improved the immune response and antioxidant capacity of sows and increased the daily BW gain of their offspring.

## 1. Introduction

The reproductive performance of sows is a very important economic indicator [1]. During gestation and lactation, sows may be exposed to various environmental and physiological factors, such as high temperatures and infections, which can negatively affect fetal development and the growth of offspring [2,3]. Furthermore, during the late gestation period until weaning, sows are subjected to severe metabolically induced oxidative stress due to the accumulation of reactive oxygen species, such as superoxide anion and hydrogen peroxide, produced by the placenta and mammary glands [4]. This oxidative stress can have a negative impact on both reproductive performance and breast milk production [5]. Previous studies have shown that oxidative stress in sows can have negative effects on litter size, live litter size and litter weight gain [6,7]. Additionally, oxidative stress can lead to reduced feed intake, resulting in decreased milk production and greater loss of body condition [8]. Antibodies are not transferred through the placenta, so piglets must obtain immunoglobulins through breast milk after birth to boost their immunity. Colostrum is the first milk secreted by sows within 24 h after giving birth [9]. It contains high levels of immunoglobulins and essential nutrients, such as proteins, fats, and lactose. Failure to obtain adequate nutrients and immunoglobulins can result in low immunity of the newborn piglets and increase the risk of death due to infections and diseases [9]. Furthermore, reproductive hormones, namely estrogen and progesterone, are essential for maintaining pregnancy [10]. A deficiency of these hormones can lead to fetal loss and reduced reproductive performance in mammals [11]. 

Puerarin, also known as daidzein 8-C-glucoside, is a major phytoestrogen found in *Pueraria mirifica*, a wild leguminous trailing plant. It is one of the main isoflavone components of *Pueraria mirifica* root [12]. *Pueraria mirifica* root has a long history of use in traditional medicine and is a common herbal remedy for fever, diarrhea, diabetes, cardiovascular disease, and cerebrovascular disease [13,14]. Pharmacological studies in vivo, using mice as a model, have demonstrated that puerarin exhibits anti-inflammatory [15], antioxidant [16], anti-apoptotic [17], and anti-myocardial fibrosis effects [12]. Puerarin showed antioxidant effects by regulating the expression of the NF-E2 p45-related factor 2 (Nrf2) pathway and antioxidant enzymes [16]. Furthermore, Li et al. [18] demonstrated that soy flavonoids enhanced sow reproductive performance by modifying serum hormones, augmenting antioxidant capacity, and regulating crucial functional genes in the placenta. Research has demonstrated that supplementing sow diets with soy isoflavones can enhance sow reproductive performance and increase the average daily weight gain of offspring [19]. Therefore, it is conjectured that puerarin, with its isoflavonoid properties, may have a positive effect on the reproductive performance and antioxidant properties in sows. No studies have been reported on the addition of puerarin to sow diets. The main objective of this study was to examine the impact of dietary supplementation of puerarin on the reproductive performance, antioxidant properties, anti-inflammatory effects, and offspring growth performance of sows with varying parities.

## 2. Materials and Methods 

The animal study protocol was approved by the Institutional Review Board (or Ethics Committee) of Southwest University of Science and Technology (protocol code SM20220214, 1 December 2022).

### 2.1. Animals and Experimental Design

The objective of this study was to examine the impact of puerarin supplementation on the reproductive performance of sows with different parities and the growth performance of their offspring. In this study, 240 sows were selected from the gestation farm of Sichuan Dekon Group Co. There were 80 gilts, 80 one-parity sows, and 80 sows with two parities or more. Before estrous, all sows were confined to pens measuring 2.25 m × 0.7 m. Once the sows entered heat, they were inseminated twice with fresh semen from three Duroc boars, each containing 3 × 10^9^ sperms. After mating, they were randomly assigned to the control and puerarin groups according to parity. The puerarin group’s diet was supplemented with 1 g/kg of puerarin. The dry powder of puerarin was gradually diluted. Puerarin (99%) was purchased from Yunnan BioBioPha Co., Ltd. (Kunming, Yunnan Province, China). The daily intake amount of puerarin was based on the relevant literature on mice [20] and laying hens [21]. Dietary composition (Table 1) during gestation and lactation was in accordance with National Research Council (2012) recommendations [22] From gestation until 1 week prior to parturition, 2.4 kg of ration was provided per day (08:00 and 16:00). The temperature of the gestation house was controlled at 20–24 °C. Before being transferred to the farrowing stalls, each sow was placed in an individual confinement pen. One week prior to parturition, pregnant sows were transferred to two separate farrowing stalls according to treatment and parities. The dimensions of the farrowing bed were 2.13 m × 1.52 m, while the sow restriction pen had a width of 0.56 m. From transfer to the farrowing house until delivery, 2.6 kg of ration was provided per day (08:00 and 16:00). On the first day after delivery, 0.5 kg of diet was provided, which was increased gradually by 1 Kg per day until maximum feed intake was achieved. Temperature in the farrowing room was maintained at 20–23 °C, and piglets were provided heat using a 250 W heat lamp. Adequate water was provided to the sows and piglets throughout the trial. All piglets were exclusively breastfed and were not provided with any additional feed or diets. At 24 h after giving birth, piglets from sows in the same treatment group, with the same parity and farrowing house, were cross-fostered. Routine procedures for lactating piglets included tooth clamping, tail removal, subcutaneous injection of iron dextran, and immunization. Piglets were weaned on day 21 after farrowing.

### 2.2. Sample Collection

Sows that were infertile, sick, abortive, and those with less than six litters were recorded separately for culling. Backfat thickness was measured using a digital backfat meter (Renco, Minneapolis, MN, USA) at 60–80 mm from the level of the last rib in the midline of the body on days 1, 30, 60, and 90 of gestation, and at farrowing. The total born, born alive, stillbirths, and mummies per pig were recorded. The litter weight was measured 1 h after birth of the last piglet. The coefficient of variation (CV) within the litter was used to determine uniformity of the neonates. Body weight (BW) per piglet and litter weaning weight were recorded after cross-fostering and again at the end of lactation (day 21). Colostrum was collected by hand-milking without the use of oxytocin, 1 h after the last piglet was born. Milk was collected on days 7 and 21 of lactation. Colostrum and regular milk were collected from the anterior, middle, and posterior parts of the mammary gland and then mixed separately. Milk samples were stored at −20 °C until further analysis. Blood was collected from the sow’s ear vein on days 1 and 21 of lactation. Approximately 20 mL of blood from each sow was collected into two no anticoagulant tubes and two heparinized tubes (Becton Dickinson and Company, Franklin Lakes, NJ, USA). At birth, one piglet per sow was used to collect umbilical cord blood into two no anticoagulant tubes. Non-anticoagulated blood samples were centrifuged at 3000× *g* and 4 °C for 15 min. The supernatant was used to obtain serum, which was then stored at −80 °C for future analysis. 

### 2.3. Determination of Milk Composition

The composition of milk (lipids, crude protein, lactose, and solids-not-fat) was determined by an automatic milk composition analyzer (Milko-Scan 134 A/B, Foss Company, Hillerød, Denmark). The study estimated the total antioxidant capability (T-AOC); the activities of superoxide dismutase (SOD), catalase (CAT), and glutathione peroxidase (GSH-Px); and malonaldehyde (MDA) content in both serum and milk using commercial kits (Nanjing Jiancheng Biology Co., Ltd., Nanjing, China) and following the manufacturer’s protocols. A V1600 Split Beam Visible Spectrophotometer (Meipuda Co., Shanghai, China) was used for the analysis. The concentrations of immunoglobulin A (IgA), immunoglobulin G (IgG), and immunoglobulin M (IgM) in milk were determined using enzyme-linked immunosorbent assay (ELISA) kits (Nanjing Jiancheng Biology Co., Ltd.), according to the manufacturer’s instructions.

### 2.4. Determination of Serum and Plasma Composition

The plasma concentrations of glucose (GLU), triacylglycerols (TAG), and urea were determined using specific assay kits from the Nanjing Jiancheng Bioengineering Institute in Nanjing, China, following the manufacturer’s instructions. Plasma activities of alanine aminotransferase (ALT), aspartate aminotransferase (AST), alkaline phosphatase (ALP), and gamma-glutamyl transpeptidase (γ-GGT) as well as the concentration of total bilirubin (TBIL) were measured using corresponding commercial kits (Sichuan Maker Biotechnology Inc., Chengdu, China) and an automatic biochemical analyzer (Hitachi 7020, Hitachi High-Technologies Corporation, Tokyo, Japan).

Serum concentrations of interleukin-6 (IL-6), interleukin-10 (IL-10), tumor necrosis factor-α (TNF-α), and interferon-γ (INF-γ) were measured in sows and piglets using the ELISA kit from the Jiancheng Institute of Biological Technology in Nanjing, China. The cytokine analysis followed the manufacturer’s instructions, and each sample was analyzed in duplicate.

Concentrations of IgA, IgG, and IgM were analyzed using ELISA kits validated for swine (Nanjing Jiancheng Biology Co., Ltd.) according to methods described.

The activities or contents of enzymatic and non-enzymatic antioxidants, including T-AOC, GSH-Px, MDA, CAT, and SOD, were measured in plasma samples using commercially available ELISA kits (Nanjing Jiancheng Biology Co., Ltd.) according to the manufacturer’s instructions.

### 2.5. Statistical Analysis

The Shapiro–Wilk and Levene tests were used to confirm the normality and homogeneity of variance of the data. The data were analyzed as two-factorial using the MIXED procedure in SAS 9.4 (SAS Institute Inc., Cary, NC, USA). Parity, diet, and their interaction were the fixed effects. The sow nested within a group of sows was included as a random effect. When analyzing backfat thickness and milk and serum composition, individual sows were considered as experimental units. The experimental unit for the analysis of sow reproductive performance and piglet growth performance was the pen. Tukey’s test was used to compare the differences in means between treatment groups. Statistical significance was set at *p* < 0.05. A trend was considered at 0.05 < *p* < 0.10. 

## 3. Results

### 3.1. Culling Data of Sows

Of the 240 initially selected sows, only 192 became pregnant after artificial insemination (Table 2). Among these, 14 were culled due to disease, abortion, and lameness, resulting in a total of 178 farrowing sows. However, only the performance data from 171 sows were used in the analyses, because seven sows had less than six piglets per litter (Table 2, *p* > 0.05).

### 3.2. Backfat Thickness and Reproductive Performance

We found no differences in backfat thickness among the different treatment groups at days 1, 30, 60, and 90 of gestation and at farrowing (Table 3, *p* > 0.05). Dietary supplementation with puerarin had no significant effect on reproductive performance of the sows, and no interaction effects were observed (Table 4, *p* > 0.05). We found a significant effect of parity on the born alive, litter weight at birth, and intra-litter CVs (Table 4, *p* < 0.05).

### 3.3. Growth Performance of Suckling Piglets

The daily BW gain of piglets and the mean BW of piglets at weaning were significantly increased through dietary supplementation with puerarin (Table 5, *p* < 0.05). Parity tended to increase litter size, litter weight, and mean litter weight gain (Table 5, 0.05 < *p* < 0.10) and significantly affected daily piglet BW gain (Table 5, *p* < 0.05). The growth performance of suckling piglets was not affected by the interaction between diet and parity (Table 5, *p* > 0.05).

### 3.4. Milk Composition 

Milk composition in the colostrum and milk of sows was not affected by parity and the interaction of parity and diet (Table 6, *p* > 0.05). The addition of puerarin to the diet resulted in a significant increase in the fat content of colostrum and the total solids content in both colostrum and regular milk (Table 6, *p* < 0.05). The addition of puerarin to the diet resulted in a trend toward increased lactose content in colostrum (Table 6, 0.05 < *p* < 0.10). 

### 3.5. Metabolic Parameters in Plasma

Plasma metabolic parameters were not affected by interactions between diet and parity (Table 7, *p* > 0.05). The addition of puerarin to the diet reduced plasma levels of ALT, AST, and ALP in sows on lactation day and day 21 (Table 7, *p* < 0.05). As parity increased, plasma ALT concentrations decreased in sows at day 21 (Table 7, *p* < 0.05), but there was no significant effect on concentrations/activities of urea, GLU, TAG, AST, ALP, TBIL, and γ-GGT. 

### 3.6. Serum Concentrations of Cytokines

Serum concentrations of cytokines in sows and piglets were not influenced by the parity or by the interaction between parity and diet (Table 8, *p* > 0.05). Dietary supplementation with puerarin significantly increased serum IL-10 and IFN-γ levels in sows and significantly decreased serum TNF-α levels in sows and piglets (Table 8, *p* < 0.05). In addition, dietary supplementation with puerarin tended to increase serum IL-6 levels in sows and serum IL-10 levels in piglets. Dietary supplementation with puerarin tended to decrease serum IFN-γ levels in piglets (Table 8, 0.05 < *p* < 0.10).

### 3.7. Antioxidant Parameters

The concentrations/activities of antioxidant parameters in the plasma of sows were not affected by parity, and the concentrations/activities of antioxidant parameters in sow colostrum and milk were not affected by diet, parity, or their interaction (Table 9 and Table 10, *p* > 0.05). On lactation day, plasma CAT concentrations were influenced by dietary and parity interactions (Table 9, *p* < 0.05). In comparison to the controls, sows in the puerarin group had significantly higher GSH-Px concentration in plasma on lactation day but significantly lower MDA concentration (Table 9, *p* < 0.05). On day 21, the puerarin group showed a significant increase in plasma concentrations of T-AOC and GSH-Px and a significant decrease in the MDA concentration compared to the controls (Table 9, *p* < 0.05).

### 3.8. Immunoglobulins

Table 11 and Table 12 show that dietary supplementation with puerarin significantly increased IgA and IgG levels in serum, colostrum, and milk compared to controls (*p* < 0.05). The IgA levels in colostrum decreased significantly with increasing parity (*p* < 0.05), but there was no significant effect on serum and milk IgA, IgG, and IgM levels (*p* > 0.05). No interaction effect between diet and parity were observed for IgA, IgM or IgG levels in sow serum, colostrum and milk (*p* > 0.05).

## 4. Discussion

Puerarin is an isoflavone extracted from *Pueraria mirifica*. It is a soluble aromatic compound that is structurally homologous to estradiol and interacts with the estrogen signaling pathway [12]. Puerarin is considered a phytoestrogen because it is derived from a natural plant source [23,24]. Puerarin has been utilized in the treatment of various diseases owing to its anti-tumor, anti-inflammatory, and antioxidant properties [25,26,27,28]. No studies have been conducted on the addition of puerarin alone to sow diets, despite its wide range of uses. Therefore, this study investigated the effects of puerarin-supplemented diets on reproductive performance, litter growth, milk composition, metabolic parameters, cytokine concentrations, antioxidant parameters, and immunoglobulin concentrations for sows of different parities.

The inclusion of puerarin in the diet of aged laying hens has been shown to enhance egg production and improve laying performance [21]. Furthermore, a study conducted on rats found that oral administration of puerarin significantly reduced apoptosis, thereby ameliorating the decline in ovarian reserve function [20]. However, no effect of puerarin on sow reproductive performance was found. Nonetheless, an effect of isoflavones on sow reproductive performance was observed. Studies have shown that soy flavonoids and isoflavones can improve reproductive performance and antioxidant properties in sows, and growth performance of sow offspring is improved by supplementation with soya isoflavones [18,19]. In our study, dietary supplementation with puerarin did not significantly affect sow reproductive performance but significantly increased weaning weight and daily BW gain of piglets. The difference may be because, although puerarin is an isoflavone, its metabolism in the body differs somewhat from that of soy isoflavones. It should also be noted that puerarin is insoluble in water and fat [29]. This results in a low bioavailability of only 7% when administered orally [30]. Different routes of geraniol administration to sows may yield varying results. It is important to consider the method of administration, whether oral or by injection. This hypothesis requires further experimental testing.

The milk produced by the sow is the source of nutrients for piglets. Milk composition has an important effect on piglet growth [31]. The present study showed that the puerarin group significantly increased the fat and total solids content in colostrum, as well as the total solids content in milk. The piglets in the puerarin group showed better growth performance compared to the control group. This was probably due to the higher fat and solids content of the milk, which made more nutrients available to them. Research indicates that increasing colostrum intake by piglets can lead to higher weaning weights and lower mortality rates during lactation and nursing [32]. However, it is unfortunate that lactation volume data were not collected in this study. Therefore, it was not possible to investigate whether the increase in piglet daily BW gain and weaning mean BW was due to differences in lactation capacity resulting from dietary supplementation with puerarin.

The increased metabolic demands on the mother mean that more of her body’s reserves are used for body development and growth of offspring during late pregnancy and lactation [33]. The liver plays an important role in controlling systemic metabolism [34], and increased maternal metabolic stress may lead to liver injury. Liver injury leads to an increase in the accumulation of AST, ALP, ALT, and γ-GGT enzymes in the plasma [35]. The study found that the plasma levels of ALT, AST, and ALP were significantly lower in the puerarin group compared to the controls. The result indicated that the inclusion of puerarin improved the resistance of the sows’ livers to injury.

The IL-6 and TNF-α are pro-inflammatory factors that participate in acute phase protein synthesis and have been linked to the development of various infections [36]. The results indicated that sows in the puerarin group had lower levels of IL-6 and TNF-α in their serum. IL-10 is an anti-inflammatory cytokine that down-regulates the production of pro-inflammatory cells [37]. This may prevent the systemic spread of inflammation in animals [38]. The IFN-γ plays a crucial role in the immune system by enhancing the body’s immunity against pathogens [39]. The study revealed that the puerarin group had higher levels of IL-10 in their serum. Our analysis of piglet cord blood showed that the cytokine composition was similar to that in maternal serum. Serum immunoglobulin levels provide important information about the status of humoral immunity [40]. The IgA is the antibody found in tears, saliva, and mucosal secretions [41]. Furthermore, it is found in colostrum, which grants the mother passive immunity before the newborn’s immune system fully develops and matures [42]. During the secondary immune response, the primary isotype produced is IgG, which is also the most abundant monomeric immunoglobulin found in serum [43]. It binds antigens with high affinity, activates the complement cascade, and mediates antibody-dependent cellular cytotoxicity [43]. Additionally, IgG is the only isotype that crosses the placenta, providing passive immunity to the fetus [43]. The IgM primarily contributes to the primary immune response [44]. The study results indicated that the levels of IgA and IgG in the serum, colostrum, and milk of sows in the puerarin group were significantly higher. However, there was no significant change in IgM levels. Taken together, these results suggest that the dietary addition of puerarin may have resulted in a stronger immune response in the sows, with improved immunity and anti-inflammatory capacity. In vivo studies in mice similarly found that puerarin attenuated colonic shortening, colonic pathological damage, and myeloperoxidase activity, downregulated NF-κB and pro-inflammatory mediator secretion, and significantly inhibited inflammation [16].

During pregnancy in sows, the placenta produces reactive oxygen species, including the superoxide ANION and hydrogen peroxide, which can cause oxidative stress [4]. This accumulation of excess free radicals can lead to the oxidation of lipids, proteins, and DNA, damaging cellular function [4]. If the oxidative stress state persists, it can negatively impact fetal development, potentially resulting in preterm labor, fetal growth restriction, and abortion [5]. Biochemical reactions involving SOD and GSH-Px can eliminate excess oxidative free radicals in the body, thereby reducing oxidative stress [45,46]. The superoxide ANION and hydrogen peroxide in cells can be broken down by the combined action of SOD and GSH-Px. In the body, CAT performs the same function as GSH-Px. The level of T-AOC can reflect the strength of the non-enzymatic antioxidant defense system [47]. The MDA responds to oxidative stress, which is primarily caused by the peroxidation of fat in the body [46]. Coenzyme Q10, a free radical scavenger, can improve uterine function and embryo survival, thereby enhancing sow reproductive performance [48]. A study in mice showed that puerarin displays antioxidant effects by modulating the NF-E2 p45-related factor 2 pathway and the expression of antioxidant enzymes [16]. Puerarin was found to reduce the frequency of hydrogen peroxide-induced micronucleus and DNA damage in a study evaluating its potential genotoxicity in mammalian cells (human lymphocytes and V79 cells) [49]. This research measured T-AOC, SOD, CAT, GSH-Px, and MDA in the plasma and milk of sows to investigate their redox status. In comparison to the controls, sows in the puerarin group had significantly higher GSH-Px activity in plasma on lactation day but significantly lower MDA concentration. On day 21, the puerarin group showed a significant increase in the plasma levels of T-AOC and GSH-Px and a significant decrease in MDA concentration compared to controls. The results indicated that a continuous supply of puerarin can significantly increase the antioxidant capacity of sows. Puerarin was significantly effective in ameliorating lead-induced histological changes and inhibiting lead-induced apoptosis in rat liver cells, which may be related to its antioxidant properties [50]. Therefore, the decrease in plasma levels of ALT, AST, and ALP in the puerarin group in the present study may be due to the improved antioxidant properties of the sows as caused by puerarin.

## 5. Conclusions

The addition of 1 g/kg puerarin to the diet did not significantly affect the reproductive performance of sows but improved immune response, antioxidant capacity, and total milk solids content, which in turn improved piglet growth performance.

## Figures and Tables

**Table 1:** Basic composition of the diets.

Item	Gestation	Lactation
Ingredients (g/kg)		
Corn	600	621
Soybean meal, 43% CP	125	220
Wheat bran, 15% CP	200	50
Fish meal, 62% CP	-	25
Soybean oil	10	20
L-lysine, 78%	1.5	1.0
L-threonine, 98.5%	-	0.5
Salt	4	4
Limestone	8	4
Calcium phosphate	10	13
Choline chloride, 50%	1.5	1.5
Vitamin and trace mineral premix ^1^	40	40
Total	1000	1000
Calculated composition (%)		
DE (kcal/kg)	3233	3365
CP	13.43	16.78
Ca	0.63	0.69
P	0.62	0.67
Ca:P	1.02	1.03

^1^ The premix supplied the following vitamins and trace minerals per kilogram of diet: Cu, 20 mg; I, 0.3 mg; Mn, 40 mg; Se, 0.3 mg; Fe, 165 mg; Zn, 165 mg; Cr, 0.3 mg; 25,000 IU vitamin A; 5000 IU vitamin D_3_; 12.5 IU vitamin E; 2.5 mg vitamin K; 1 mg vitamin B_1_; 8 mg vitamin B_2_; 3 mg vitamin B_6_; 0.015 mg vitamin B_12_; 17.5 mg niacin; 12.5 mg pantothenic acid; 0.25 mg folacin.

**Table 2:** Culling data of sows.

Diets	CON			PUE		
Parities	0	1	2+	0	1	2+
Initial	40	40	40	40	40	40
Concepted	31	34	30	32	35	30
Total farrowed	30	31	29	28	31	29
Farrowed effective	29	30	28	28	28	28
Total culls	11	10	12	12	12	12
Percentage culls (%)	27.5	25.0	30.0	30.0	30.0	30.0

CON, control diet; PUE, control diet supplemented with 1 g/kg puerarin.

**Table 3:** Backfat thickness of sows during gestation.

Diets	CON			PUE			SD	*p*-Value		
Parities	0	1	2+	0	1	2+		D	P	D × P
No. of sows	29	30	28	28	28	28	-	-	-	-
Backfat thickness (mm)										
Day 1	14.69	14.66	14.70	14.80	14.72	14.64	1.095	ns	ns	ns
Day 30	15.34	15.51	15.67	15.74	15.87	15.78	1.187	ns	ns	ns
Day 60	16.58	16.81	16.54	16.57	16.65	16.62	1.185	ns	ns	ns
Day 90	16.83	16.92	16.91	16.79	16.93	16.95	1.133	ns	ns	ns
At farrowing	17.31	17.48	17.42	17.48	17.49	17.44	1.139	ns	ns	ns

CON, control diet; PUE, control diet supplemented with 1 g/kg puerarin; D, diet; P, parity; D × P, interaction between parity and diet. ns, non-significant, *p* > 0.10. Trends were considered at 0.05 < *p* < 0.1.

**Table 4:** Effect of dietary puerarin supplementation on reproductive performance of sows.

Diets	CON			PUE			SD	*p*-Value		
Parities	0	1	2+	0	1	2+		D	P	D × P
No. of sows	29	30	28	28	28	28	-	-	-	-
Total born	13.5	14.3	14.2	13.4	14.3	14.4	2.229	ns	0.08	ns
Born alive	12.4	13.1	13.5	12.6	13.3	13.4	1.843	ns	*	ns
Stillborn	1.07	1.17	0.64	0.82	0.93	1.00	1.034	ns	ns	ns
Number of mummies	0.28	0.30	0.29	0.21	0.25	0.18	0.440	ns	ns	ns
Mean body weight at birth (kg)	1.49	1.53	1.58	1.43	1.52	1.57	0.292	ns	ns	ns
Litter weight at birth (kg)	18.6	20.0	21.1	18.1	20.5	21.0	4.743	ns	*	ns
Intra-litter CV (%)	16.16 ^c^	17.18 ^b^	18.14 ^a^	16.20 ^c^	16.94 ^b^	18.02 ^a^	0.597	ns	*	ns

CON, control diet; PUE, control diet supplemented with 1 g/kg puerarin; CV, coefficient of variation; D, diet; P, parity; D × P, interaction between parity and diet. *, *p* < 0.05; ns, non-significant, *p* > 0.10. Trends were considered at 0.05 < *p* < 0.1. ^a, b, c^ Means in the same row with different superscripts differ significantly (*p* < 0.05).

**Table 5:** Effect of dietary puerarin supplementation on growth performance of suckling piglets.

Diets	CON			PUE			SD	*p*-Value		
Parities	0	1	2+	0	1	2+		D	P	D × P
No. of sows	29	30	28	28	28	28	-	-	-	-
Litter size										
After cross-foster	12.1	12.6	12.9	12.3	12.8	12.9	1.596	ns	0.051	ns
At weaning	12.0	12.4	12.7	12.0	12.6	12.5	1.492	ns	0.074	ns
Mean BW of piglet (kg)										
After cross-foster	1.70	1.69	1.71	1.76	1.74	1.73	0.362	ns	ns	ns
At weaning	6.89	6.95	6.97	7.08	7.23	7.24	0.658	*	ns	ns
Litter weight (kg)										
After cross-foster	13.8	14.3	14.6	14.0	14.6	14.6	1.707	ns	0.082	ns
At weaning	82.6	86.5	88.8	85.5	91.1	90.7	14.35	ns	0.081	ns
Daily BW gain of piglets (g/d)	259.4 ^b^	262.8 ^ab^	263.1 ^ab^	265.9 ^ab^	274.8 ^a^	275.3 ^a^	15.3	*	*	ns
Mean litter weight gain (kg)	3.44	3.61	3.71	3.58	3.83	3.81	0.652	ns	0.092	ns

CON, control diet; PUE, control diet supplemented with 1 g/kg puerarin; BW, body weight; D, diet; P, parity; D × P, interaction between parity and diet. *, *p* < 0.05; ns, non-significant, *p* > 0.10. Trends were considered at 0.05 < *p* < 0.1. ^a, b^ Means in the same row with different superscripts differ significantly (*p* < 0.05).

**Table 6:** Effect of dietary puerarin supplementation on colostrum and milk composition of sows.

Diets	CON			PUE			SEM	*p*-Value		
Parities	0	1	2+	0	1	2+		D	P	D × P
No. of sows	12	12	12	12	12	12	-	-	-	-
Colostrum (%)										
Fat	4.49 ^b^	5.07 ^ab^	4.86 ^ab^	5.43 ^ab^	5.67 ^a^	5.70 ^a^	0.268	*	ns	ns
Lactose	2.68	2.76	2.82	2.96	3.20	3.21	0.236	0.059	ns	ns
True protein	15.94	16.13	15.84	15.96	16.08	16.09	0.168	ns	ns	ns
Urea	64.53	65.80	65.46	66.74	66.78	66.71	1.033	ns	ns	ns
Total solids	23.33	23.12	23.38	24.63	24.26	24.28	0.474	*	ns	ns
Milk (%)										
Fat	8.48	8.77	8.65	8.41	8.13	8.59	0.387	ns	ns	ns
Lactose	5.49	5.37	5.62	5.42	5.32	5.49	0.262	ns	ns	ns
True protein	4.74	4.79	4.34	4.70	4.22	4.62	0.241	ns	ns	ns
Urea	53.4	51.9	52.6	52.9	52.4	53.5	0.574	ns	ns	ns
Total solids	18.56	18.75	18.80	19.78	19.43	19.90	0.424	*	ns	ns

CON, control diet; PUE, control diet supplemented with 1 g/kg puerarin; D, diet; P, parity; D × P, interaction between parity and diet. *, *p* < 0.05; ns, non-significant, *p* > 0.10. Trends were considered at 0.05 < *p* < 0.1. ^a, b^ Means in the same row with different superscripts differ significantly (*p* < 0.05).

**Table 7:** Effect of dietary puerarin supplementation on metabolic parameters in plasma of sows.

Diets	CON			PUE			SEM	*p*-Value		
Parities	0	1	2+	0	1	2+		D	P	D × P
No. of sows	12	12	12	12	12	12	-	-	-	-
Lactation day (Day 1)										
Urea (mmol/L)	4.16	3.95	4.19	4.02	4.23	4.03	0.174	ns	ns	ns
GLU (mmol/L)	4.12	3.91	3.90	3.94	4.06	4.17	0.205	ns	ns	ns
TAG (mmol/L)	0.22	0.29	0.24	0.21	0.27	0.31	0.042	ns	ns	ns
ALT (U/L)	28.92	27.51	29.16	26.99	25.98	26.71	0.730	*	ns	ns
AST (U/L)	35.15	34.53	33.54	33.41	32.60	32.37	0.693	*	ns	ns
ALP (U/L)	54.61 ^ab^	56.48 ^a^	56.69 ^a^	52.41 ^ab^	51.94 ^ab^	50.78 ^b^	1.097	*	ns	ns
TBIL (mmol/L)	1.85	2.13	2.15	1.80	1.92	2.05	0.166	ns	ns	ns
γ-GGT (U/L)	65.41	66.11	65.17	65.51	64.62	65.68	0.590	ns	ns	ns
Day 21										
Urea (mmol/L)	5.93	6.28	6.27	6.11	6.05	6.02	0.149	ns	ns	ns
GLU (mmol/L)	35.10	36.81	35.30	35.41	36.09	36.40	0.573	ns	ns	ns
TAG (mmol/L)	0.39	0.42	0.39	0.42	0.35	0.24	0.057	ns	ns	ns
ALT (U/L)	47.78 ^a^	48.13 ^a^	46.89 ^a^	43.89 ^ab^	43.00 ^ab^	37.92 ^b^	1.291	*	*	ns
AST (U/L)	35.42 ^a^	34.65 ^a^	35.87 ^ab^	29.92 ^ab^	30.92 ^ab^	27.33 ^b^	1.376	*	ns	ns
ALP (U/L)	39.45 ^a^	37.39 ^a^	33.37 ^a^	21.23 ^b^	23.85 ^b^	20.70 ^b^	2.372	*	ns	ns
TBIL (mmol/L)	0.62	0.50	0.44	0.52	0.43	0.59	0.085	ns	ns	ns
γ-GGT (U/L)	48.47	39.47	47.24	47.82	44.27	47.11	4.412	ns	ns	ns

CON, control diet; PUE, control diet supplemented with 1 g/Kg puerarin; GLU, glucose; TAG, triacylglycerols; ALT, alanine aminotransferase; AST, aspartate aminotransferase; ALP, alkaline phosphatase; TBIL, total bilirubin; γ-GGT, gamma-glutamyl transpeptidase; D, diet; P, parity; D × P, interaction between parity and diet. *, *p* < 0.05; ns, non-significant, *p* > 0.10. Trend was considered at 0.05 < *p* < 0.1. ^a, b^ Means in the same row with different superscripts differ significantly (*p* < 0.05).

**Table 8:** Effect of dietary puerarin supplementation on serum concentrations of cytokines in sows and piglets on day 1.

Diets	CON			PUE			SEM	*p*-Value		
Parities	0	1	2+	0	1	2+		D	P	D × P
No. of sows	12	12	12	12	12	12	-	-	-	-
Sows (ng/L)										
IL-6	112.8	116.5	120.8	117.1	117.6	112.7	2.701	0.06	ns	ns
IL-10	171.0 ^b^	175.9 ^ab^	174.9 ^ab^	183.2 ^ab^	189.5 ^a^	185.8 ^ab^	21.8	*	ns	ns
TNF-α	21.21 ^ab^	22.31 ^a^	21.07 ^ab^	16.55 ^b^	17.90 ^ab^	16.19 ^b^	1.278	*	ns	ns
IFN-γ	94.20	93.61	94.21	99.02	100.5	95.92	2.238	*	ns	ns
Piglets (ng/L)										
IL-6	164.7	160.3	161.0	160.7	161.3	160.8	3.380	ns	ns	ns
IL-10	49.80	53.27	52.79	55.13	54.43	54.78	2.750	0.093	ns	ns
TNF-α	37.73 ^ab^	40.97 ^a^	39.28 ^a^	31.49 ^b^	35.97 ^ab^	33.46 ^ab^	1.815	*	ns	ns
IFN-γ	103.9	108.1	105.7	103.9	102.2	100.0	2.656	0.07	ns	ns

CON, control diet; PUE, control diet supplemented with 1 g/kg puerarin; IL-6, interleukin-6; IL-10, interleukin-10; TNF-α, tumor necrosis factor-α; INF-γ, interferon-γ; D, diet; P, parity; D × P, interaction between parity and diet. *, *p* < 0.05; ns, non-significant, *p* > 0.10. Trends were considered at 0.05 < *p* < 0.1. ^a, b^ Means in the same row with different superscripts differ significantly (*p* < 0.05).

**Table 9:** Effect of dietary puerarin supplementation on concentrations and activities of antioxidant parameters of sow plasma.

Diets	CON			PUE			SEM	*p*-Value		
Parities	0	1	2+	0	1	2+		D	P	D × P
No. of sows	12	12	12	12	12	12	-	-	-	-
Lactation day (Day 1)										
T-AOC (U/mL)	1.99	1.94	1.91	2.40	2.18	2.12	0.178	0.052	ns	ns
GSH-Px (U/mL)	1006	996	989	1037	1027	1023	18.92	*	ns	ns
CAT (U/mL)	36.3	32.8	38.8	37.1	35.1	32.9	1.643	ns	ns	*
MDA (nmol/mL)	4.39	4.36	3.89	3.80	3.58	3.62	0.245	*	ns	ns
SOD (U/mL)	130.5	124.5	128.0	136.9	132.1	127.9	4.100	ns	ns	ns
Day 21										
T-AOC (U/mL)	1.29 ^ab^	1.23 ^b^	1.02 ^b^	2.00 ^a^	1.80 ^ab^	1.86 ^ab^	0.174	*	ns	ns
GSH-Px (U/mL)	873.1	827.7	838.7	900.6	902.3	893.6	18.48	*	ns	ns
CAT (U/mL)	33.06	34.99	36.89	32.73	32.10	35.65	1.670	ns	ns	ns
MDA (nmol/mL)	5.59 ^ab^	6.03 ^a^	5.49 ^ab^	5.25 ^ab^	5.08 ^b^	5.05 ^b^	0.229	*	ns	ns
SOD (U/mL)	100.9	96.1	102.3	103.8	103.3	102.1	2.672	ns	ns	ns

CON, control diet; PUE, control diet supplemented with 1 g/kg puerarin; T-AOC, total antioxidant capability; GSH-Px, glutathione peroxidase; MDA, malondialdehyde; CAT, catalase; SOD, superoxide dismutase; D, diet; P, parity; D × P, interaction between parity and diet. *, *p* < 0.05; ns, non-significant, *p* > 0.10. Trends were considered at 0.05 < *p* < 0.1. ^a, b^ Means in the same row with different superscripts differ significantly (*p* < 0.05).

**Table 10:** Effect of dietary puerarin supplementation on concentrations and activities of antioxidant parameters of sow colostrum and milk.

Diets	CON			PUE			SEM	*p*-Value		
Parities	0	1	2+	0	1	2+		D	P	D × P
No. of sows	12	12	12	12	12	12	-	-	-	-
Colostrum										
T-AOC (U/mL)	5.63	5.91	5.49	5.55	5.78	5.34	0.258	ns	ns	ns
GSH-Px (U/mL)	158.3	165.4	165.7	165.1	166.5	164.0	6.028	ns	ns	ns
CAT (U/mL)	38.33	40.11	40.64	39.05	38.21	38.08	1.773	ns	ns	ns
MDA (nmol/mL)	11.88	12.23	12.24	11.73	10.95	12.54	0.539	ns	ns	ns
SOD (U/mL)	333.5	344.3	347.9	343.4	344.2	339.8	7.872	ns	ns	ns
Day 21 of milk										
T-AOC (U/mL)	5.52	5.70	5.39	5.77	5.76	5.80	0.260	ns	ns	ns
GSH-Px (U/mL)	61.1	63.9	60.0	66.2	69.9	63.0	2.921	0.051	ns	ns
CAT (U/mL)	39.1	35.2	39.1	36.1	38.4	38.8	1.934	ns	ns	ns
MDA (nmol/mL)	7.59	7.34	7.60	7.18	7.86	7.45	0.263	ns	ns	ns
SOD (U/mL)	279.1	286.6	275.8	271.8	280.6	281.9	7.266	ns	ns	ns

CON, control diet; PUE, control diet supplemented with 1 g/kg puerarin; T-AOC, total antioxidant capability; GSH-Px, glutathione peroxidase; MDA, malondialdehyde; CAT, catalase; SOD, superoxide dismutase; D, diet; P, parity; D × P, interaction between parity and diet. ns, non-significant, *p* > 0.10. Trends were considered at 0.05 < *p* < 0.1.

**Table 11:** Effect of dietary puerarin supplementation on immunoglobulins in sow serum.

Diets	CON			PUE			SEM	*p*-Value		
Parities	0	1	2+	0	1	2+		D	P	D × P
No. of sows	12	12	12	12	12	12	-	-	-	-
Lactation day (mg/mL, Day 1)										
IgA	0.59 ^b^	0.51 ^b^	0.48 ^b^	1.04 ^a^	0.98 ^a^	1.10 ^a^	0.087	*	ns	ns
IgG	1.92 ^b^	2.02 ^ab^	2.10 ^ab^	2.32 ^ab^	2.55 ^ab^	2.63 ^a^	0.163	*	ns	ns
IgM	0.13	0.17	0.12	0.15	0.14	0.14	0.024	ns	ns	ns
Day 21 (mg/mL)										
IgA	0.51 ^b^	0.53 ^b^	0.56 ^b^	1.24 ^a^	1.26 ^a^	1.29 ^a^	0.082	*	ns	ns
IgG	0.74 ^b^	0.77 ^b^	0.77 ^b^	1.55 ^a^	1.57 ^a^	1.56 ^a^	0.083	*	ns	ns
IgM	0.48	0.56	0.47	0.46	0.55	0.59	0.080	ns	ns	ns

CON, control diet; PUE, control diet supplemented with 1 g/kg puerarin; IgA, immunoglobulin A; IgG, immunoglobulin G; IgM, immunoglobulin M; D, diet; P, parity; D × P, interaction between parity and diet. *, *p* < 0.05; ns, non-significant, *p* > 0.10. Trends were considered at 0.05 < *p* < 0.1. ^a, b^ Means in the same row with different superscripts differ significantly (*p* < 0.05).

**Table 12:** Effect of dietary puerarin supplementation on immunoglobulins in colostrum and milk of sows.

Diets	CON			PUE			SEM	*p*-Value		
Parities	0	1	2+	0	1	2+		D	P	D × P
No. of sows	12	12	12	12	12	12	-	-	-	-
Colostrum (mg/mL)										
IgA	4.87 ^c^	4.51 ^c^	4.04 ^c^	10.20 ^a^	9.02 ^ab^	8.27 ^b^	0.275	*	*	ns
IgG	21.52 ^b^	21.16 ^c^	20.86 ^c^	24.80 ^a^	24.70 ^a^	23.91 ^ab^	0.520	*	ns	ns
IgM	1.99	1.84	2.01	197	1.67	1.87	0.181	ns	ns	ns
Day 21 of milk (mg/mL)										
IgA	0.54 ^b^	0.55 ^b^	0.53 ^b^	1.04 ^a^	1.00 ^a^	1.03 ^a^	0.077	*	ns	ns
IgG	1.20	1.04	1.12	1.48	1.43	1.39	0.159	*	ns	ns
IgM	0.18	0.14	0.16	0.18	0.18	0.17	0.029	ns	ns	ns

CON, control diet; PUE, control diet supplemented with 1 g/kg puerarin; IgA, immunoglobulin A; IgG, immunoglobulin G; IgM, immunoglobulin M; D, diet; P, parity; D × P, interaction between parity and diet. *, *p* < 0.05; ns, non-significant, *p* > 0.10. Trends were considered at 0.05 < *p* < 0.1. ^a, b, c^ Means in the same row with different superscripts differ significantly (*p* < 0.05).

## Data Availability

The data presented in this study are available on request from the corresponding author.
